# Effects of Arachidonic Acid Metabolites on Cardiovascular Health and Disease

**DOI:** 10.3390/ijms222112029

**Published:** 2021-11-06

**Authors:** Yan Zhou, Haroon Khan, Jianbo Xiao, Wai San Cheang

**Affiliations:** 1 Institute of Chinese Medical Sciences, State Key Laboratory of Quality Research in Chinese Medicine, University of Macau, Avenida da Universidade, Taipa, Macau 999078, China; yc07517@um.edu.mo; 2 Department of Pharmacy, Abdul Wali Khan University, Mardan 23200, Pakistan; haroonkhan@awkum.edu.pk; 3 Department of Analytical Chemistry and Food Science, Faculty of Food Science and Technology, University of Vigo, 36310 Vigo, Spain; jianboxiao@uvigo.es; 4 International Research Center for Food Nutrition and Safety, Jiangsu University, Zhenjiang 212013, China

**Keywords:** arachidonic acid, prostaglandin, cardiovascular disease, vascular tone

## Abstract

Arachidonic acid (AA) is an essential fatty acid that is released by phospholipids in cell membranes and metabolized by cyclooxygenase (COX), cytochrome P450 (CYP) enzymes, and lipid oxygenase (LOX) pathways to regulate complex cardiovascular function under physiological and pathological conditions. Various AA metabolites include prostaglandins, prostacyclin, thromboxanes, hydroxyeicosatetraenoic acids, leukotrienes, lipoxins, and epoxyeicosatrienoic acids. The AA metabolites play important and differential roles in the modulation of vascular tone, and cardiovascular complications including atherosclerosis, hypertension, and myocardial infarction upon actions to different receptors and vascular beds. This article reviews the roles of AA metabolism in cardiovascular health and disease as well as their potential therapeutic implication.

## 1. Introduction

Polyunsaturated fatty acids (PUFAs) have long been considered to have positive effects on cardiovascular health [1]. A meta-analysis study demonstrated a reduction in the risk of cardiovascular events by increasing PUFAs intake [2]. It is noteworthy that arachidonic acid (AA), one of the most abundant PUFAs in the human body, is a long-chain polyunsaturated omega-6 fatty acid containing 20 carbon atoms and 4 double bonds (20:4, n-6), and its role in cardiovascular function has been revealed in recent studies [3,4,5,6]. AA has several physiological functions: as a constituent in the phospholipid bilayer of cell membranes, as a precursor for a crucial group of biologically active compounds known as eicosanoids (C_20_ unsaturated lipids), as a regulator of gene expression, as inflammatory intermediator, and as vasodilator/vasoconstrictor [7,8,9]. Its mobility and flexibility are attributed to its four *cis* double bonds that maintain the fluidity of cell membranes at physiological temperatures, and the double bonds can react with oxygen to form eicosanoids and isoprostanes through enzymatic and non-enzymatic mechanisms, respectively [10,11]. Endogenous AA is mainly released through cell membrane phospholipids to produce a myriad of bioactive derivatives, eicosanoids, which can be metabolized by three different enzyme systems, including cyclooxygenase (COX), cytochrome P450 (CYP) enzymes, and lipid oxygenase (LOX) [12]. The oxidized lipid molecules participate in a variety of physiological and pathological functions. Upon binding to corresponding receptors and triggering the downstream signaling pathways in different tissues, AA metabolites play differential roles to control important cellular processes, such as cell apoptosis, cell proliferation, metabolism, and vascular function [7,13], and they are also related to many chronic diseases, especially cardiovascular diseases (CVD) [14,15].

CVD is a disease with high mortality and recurrence rate worldwide [16]. CVD is a hemorrhagic or ischemic disease of blood vessels caused by atherosclerosis and encompasses several entities such as hypertension, pulmonary arterial hypertension (PAH), cardiac hypertrophy, and heart failure [17]. The main risk factor for CVD is metabolic disorder including obesity and diabetes [18]. Despite the recent advances in drug and surgical treatment [19], CVD is still a common and progressive disease that threatens human health. Studies have shown that AA metabolites play an important role in cardiovascular health and disease mechanism, especially related to inflammation and atherosclerosis [15]. AA metabolites regulate the complex vascular functions in the human body and also play a role in the treatment of CVD. The current article reviews the comprehensive involvement of AA metabolites in cardiovascular function, from the differential signaling pathways mediated to their potential therapeutic implications.

## 2. Food Sources of Arachidonic Acid

**Figure 1:**
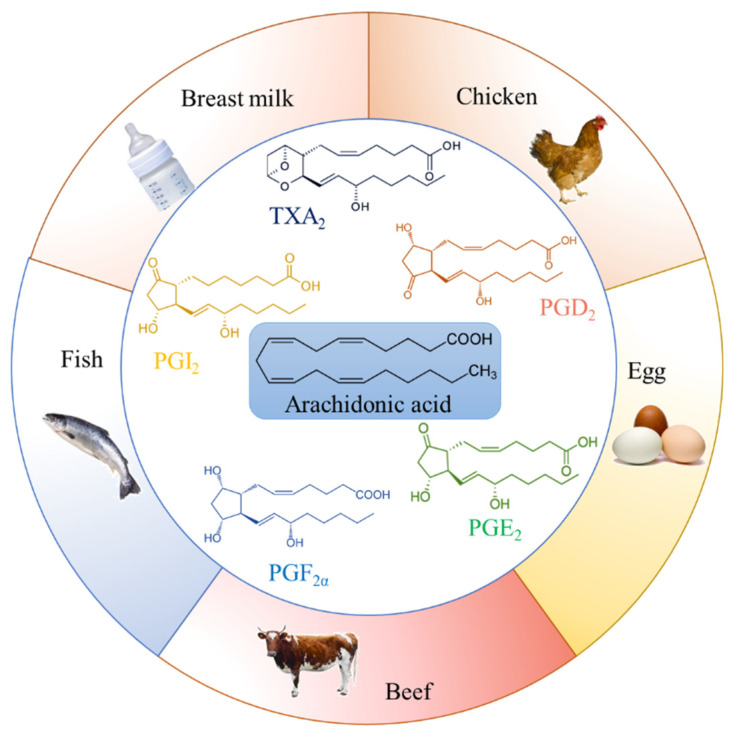
Food items rich in arachidonic acid. Chicken and chicken-mixed dishes, eggs, beef and beef products, pork and pork products, invertebrates and fish, breast milk and infant formula milk powder provide the most arachidonic acid intake to humans. Prostaglandin (PG)D_2_, PGE_2_, PGF_2α_, prostacyclin (PGI_2_), and thromboxanes (TX)A_2_ are major arachidonic acid metabolites. These polyunsaturated fatty acids play an important role in their growth and reproduction.

As an important fatty acid for human growth and development, AA can be obtained from various types of food, especially meat and fish products. Food items rich in AA can be seen in Figure 1. According to previous investigations, chicken and chicken-mixed dishes provide the most AA intake to Americans, while eggs, beef and beef products, as well as pork and pork products, are other major contributors of AA from the American diet [20]. Pork provides the most abundant AA intake to South Korea diet, accounting for 70%, followed by eggs [21]. Moreover, invertebrates and fish are rich sources of omega-3 fatty acids and also rich in AA [22]. These PUFAs play important roles in their growth and reproduction, especially in fish; therefore, AA is highly retained in phospholipids of all species [23,24]. The intake of pre-formed AA in food is an important cause of circulating AA [25]. However, previous studies mainly show the dietary content and human intake of AA in America and South Korea. Data on dietary AA from other countries are lacking, and further studies are needed to explore and provide new insights into the relationship between dietary AA with cardiovascular health.

On the other hand, AA can be converted from linoleic acid even though such endogenous conversion from linoleic acid to AA is generally poor [26]. Linoleic acid and gamma-linolenic acid are the major dietary PUFAs in common diets, and when we consume large amounts of them in our diet, our body can make AA from them [27]. Linoleic acid is broken down into gamma-linolenic acid, which is converted to AA subsequently. Linoleic acid is present in nuts, seeds, and vegetable oils including canola, corn, safflower, and sunflower oils, whereas gamma-linolenic acid is found in black current seed oil, borage oil, evening primrose oil, oats, and spirulina [28]. Around 5% to 10% of linoleic acid is taken up in phospholipid membranes and then converted to AA and bioactive lipid products [29]. Notably, improper intake of PUFAs can lead to chronic diseases such as CVD and metabolic diseases [30]. Notably, AA is a natural component found in breast milk, produced by the conversion of linoleic acid and alpha-linolenic acid of PUFAs [31], having a positive effect on growth and development, such as the brain, retina, and other nervous systems; AA is also added to infant formula milk powder [32]. Compared with European and American countries, the level of AA in Chinese mothers’ breast milk is significantly higher even though the intake of AA is lower [33]. The World Health Organization (WHO) recommends that the AA content should be at least 0.66% of the total fatty acids [34,35] based on the global average level of breast milk, while the French Food Safety Agency proposed a minimum AA intake of 0.5% of total fatty acids [36]. These results indicate that AA is abundant in various food items. The intake of pre-formed AA in food rather than conversion from linoleic acid appears to be a more important source of circulating AA. Thus, the impact of AA on cardiovascular functions is discussed further in the following sections.

## 3. Pathways Involved in Arachidonic Acid Metabolism

**Figure 2:**
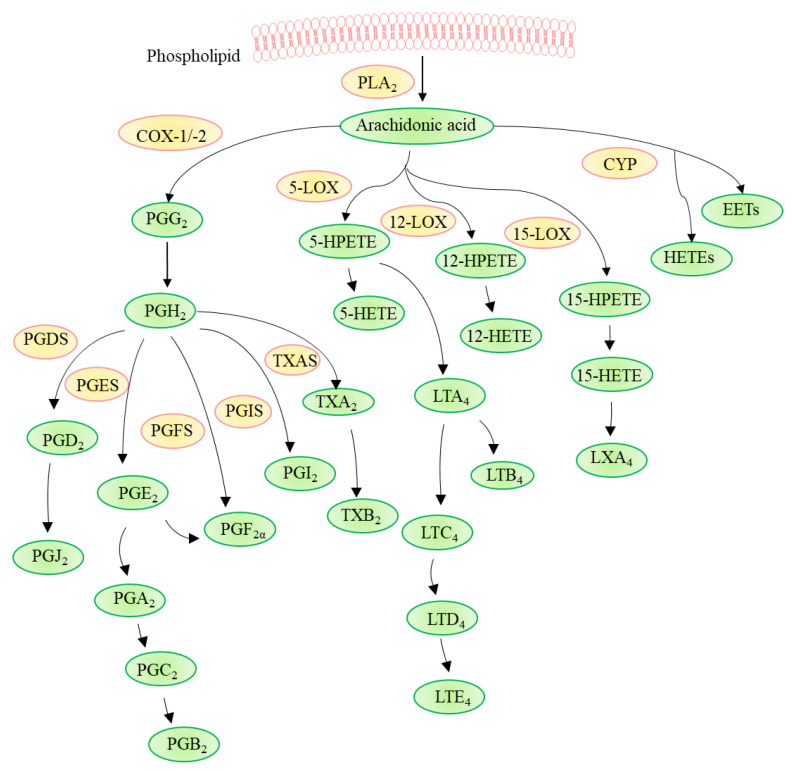
Arachidonic acid metabolism. Arachidonic acid is released from phospholipid by phospholipase A_2_ (PLA_2_) and, in turn, is metabolized through three different pathways involving cyclooxygenase (COX), cytochrome P450 (CYP) enzymes, or lipoxygenase (LOX). COX-1 and COX-2 convert arachidonic acid to prostaglandin (PG)G2 and PGH2, which are further metabolized to various prostaglandins (PGs) such as PGD_2_, PGE_2_, PGF_2α_, prostacyclin (PGI_2_), and thromboxanes (TX)A_2_ by corresponding synthases, i.e., PGD_2_ synthase (PGDS), PGE_2_ synthase (PGES), PGF_2α_ synthase (PGFS), PGI_2_ synthase (PGIS), and TXA_2_ synthase (TXAS). LOXs catalyze the dioxygenation of polyunsaturated fatty acids to their corresponding hydroperoxyeicosatetraenoic acids (HPETEs), which are subsequently converted to hydroxyeicosatetraenoic acids (HETEs), leukotrienes (LTs), and lipoxins (LXs). CYP generates HETEs and epoxyeicosatrienoic acids (EETs).

AA is released from cell membrane phospholipids primarily by phospholipase A_2_ (PLA_2_) and, in turn, metabolized through three different pathways involving cyclooxygenase (COX), cytochrome P450 (CYP) enzymes (ω-hydroxylases and epoxygenases), or lipoxygenase (LOX) (Figure 2) [37,38]. Various substances such as acetylcholine and shear stress can activate PLA_2_ and trigger the downstream mechanism in vascular endothelial cells (ECs). AA metabolism takes place in several organs including the brain, heart, kidney, liver, lung, and vasculature [12].

In the first pathway, COX, also known as prostaglandin G/H synthases (PGHS), which is present in macrophages and ECs, contributes to the production of autoregulatory and homeostatic prostanoids [13]. There are two subtypes of COX: COX-1 exists in most cells and is the primary source of prostanoids with protective functions, while COX-2 is induced by pro-inflammatory factors, growth factors, and hormones to produce prostanoids in pathological conditions such as CVD, cancer, and inflammation [39]. COX-1 and COX-2 convert AA to prostaglandin (PG)G_2_ and PGH_2_, which are further metabolized to various PGs (including PGD_2_, PGE_2_, and PGF_2α_), prostacyclin (PGI2), and thromboxane (TX)A_2_ via their respective synthases [40]. The expressions of various enzymes in different inflammatory cells determine distinct AA metabolism. For example, macrophages convert AA into PGE_2_ and TXA_2_ mainly through COX, which is involved in the development of atherosclerosis and thrombosis [41,42]. PGE2 is further hydrolyzed to produce PGA_2_, PGC_2,_ and PGB_2_ sequentially, and TXA_2_ is hydrolyzed to produce TXB_2_ [43], whereas mast cells produce PGD_2_, which is hydrolyzed to PGJ_2_ [44]. PGD_2_ plays an important protective role in cardiovascular diseases by increasing vascular permeability and blood flow, thereby resisting atherosclerosis and thrombosis [45].

In the second pathway, LOXs, including 5-LOX, 12-LOX, and 15-LOX catalyze the dioxygenation of AA to their corresponding hydroperoxyeicosatetraenoic acids (HPETEs), i.e., 5-HPETE, 12-HPETE, 15-HPETE, which are subsequently converted to hydroxyeicosatetraenoic acids (HETEs), leukotrienes (LTs), and lipoxins (LXs) [46,47]. Notably, the metabolic pathway of LOXs is associated with the progression of CVD, such as atherosclerosis [48]. In addition, 5-LOX can be expressed by different types of white blood cells to insert molecular oxygen at a specific position of AA to form 5-HPETE and LTA_4_, which are further hydrolyzed to generate LTB_4_, LTC_4_, LTD_4_, and LTE_4_ [49], and these LTs aggravate the risk of atherosclerosis or myocardial infarction [50]. The 15-LOX pathway has anti-inflammatory and pro-inflammatory effects and is also involved in the process of atherosclerosis [51]. AA generates 15-HPETE under the catalysis of this enzyme, followed by 15-HETE and LXA_4_ [52,53].

Thirdly, the CYP pathway contributes mainly to the metabolism of lipophilic xenobiotics, including drugs, fatty acids, and fat-soluble vitamins, and its expression is affected by various growth factors and hormones [54,55]. CYP is easily affected by external factors, generating various HETEs and epoxyeicosatrienoic acids (EETs) from AA [56,57]. Among them, 20-HETE and various EETs are related to the regulation of vascular tone and have cardioprotective effects [58]. The AA metabolites obtained through these three pathways are fully involved in the process of CVD, especially atherosclerosis.

AA metabolites bind to different G-protein-coupled receptors (GPCRs), mediating distinct signaling pathways. There are two PGD_2_ receptors—namely, DP1 and DP2 [59]. PGE_2_ binds to EP1, EP2, EP3, and EP4 receptors, which mediate diverse functions [60]. Furthermore, there is one receptor each for PGF_2α_, PGI_2,_ and TXA_2_ to activate FP, IP, and TP, respectively [61]. Two TP subtypes are available: TPα is present in platelets and smooth muscle cells (SMCs), whereas TPβ is present in ECs and SMCs [62,63]. Notably, cross binding of lipid mediators derived from AA to different receptors results in the versatility of signaling [64], as for example, TP can be activated by PGH_2_, PGD_2_, PGE_2_, PGF_2α_, and 20-HETE [65,66]. Additionally, LTs act on BLT1 and BLT2, which modulate chemotaxis [67]. Cysteinyl LTs (LTs having the amino acid cysteine in their structure) bind to CysLT_1_ and CysLT_2_ receptors, modulating vascular tone. Activation of CysLT_2_ results in relaxation of pulmonary arteries and increases the expression of inflammatory proteins in ECs [68]. LXs bind to formyl peptide receptor 2 (ALX/FPR2) present in leukocytes for the regulation of inflammatory responses [69].

Apart from the enzymatic actions to generate eicosanoids, AA can undergo oxidative modification to form isoprostanes through a non-enzymatic, free radical-catalyzed mechanism. These isoprostanes can serve as markers of oxidative stress and are linked to CVD, where the 8-iso-PGF_2α_ level shows a positive correlation with the presence and extent of coronary stenosis [70]. Notably, 8-iso-PGF_2α_ produced in a COX-dependent manner is associated with hypoxia-induced hyperreactivity of pulmonary arteries by activating TP receptors [71]. These AA metabolites regulate different signaling pathways by binding different receptors, thereby affecting cardiovascular functions. The upregulation and/or downregulation of the three AA metabolic pathways lead to or protect against CVDs, and such connections are evaluated in the subsequent sections.

## 4. Contribution of AA Metabolites to the Regulation of Vascular Tone

**Figure 3:**
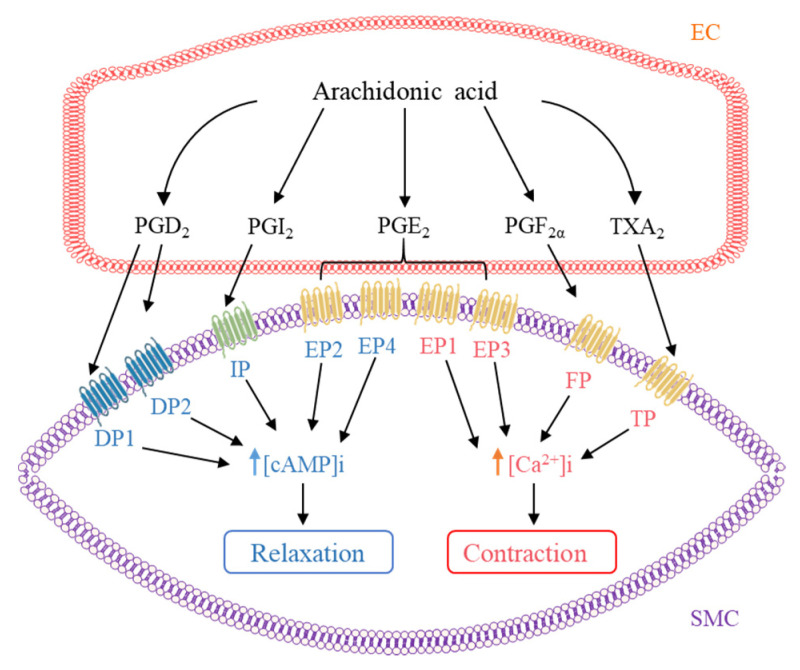
Regulation of vascular tone by arachidonic acid metabolites. Arachidonic acid metabolites produced in endothelial cell (EC) including prostaglandin (PG)D_2_, PGE_2_, PGF_2α_, prostacyclin (PGI_2_), and thromboxanes (TX)A_2_ target to their corresponding receptor in smooth muscle cell (SMC), increasing intracellular cAMP level [cAMP]i to induce relaxation or decreasing intracellular calcium level [Ca^2+^]i to induce contraction.

Various AA metabolites such as PGs and TX can act as vasodilators or vasoconstrictors to modulate vascular tone in both physiological and pathophysiological conditions (Figure 3) [72]. The same PG may induce opposite effects depending on the type of receptor it binds to in different tissues.

PGI_2_, the principal AA metabolite, is mainly produced by platelets and vascular ECs and affects cardiovascular health, inducing vasodilatation through activation of IP receptors [73]. Activation of muscarinic receptors by agonists such as acetylcholine or bradykinin triggers the production of inositol 1,4,5-triphosphate (IP_3_) and diacylglycerol (DAG) and thereby increases endothelial intracellular calcium level [Ca^2+^]i. Elevation of endothelial [Ca^2+^]i activates PLA_2_ to release free AA from cell membrane, while DAG is converted to 2-arachidonoylglycerol, which is further metabolized to generate free AA. Free AA is metabolized by COX to PGI_2_, which exerts vasodilatory responses [74]. In contrast, PGI_2_ evokes vasoconstriction when acting through TP and EP3 receptors [75]. In mice, inhibition of COX-2-dependent PGI_2_ biosynthesis results in depression of endothelial nitric oxide synthase (eNOS) and thereby reduces nitric oxide (NO) bioavailability, resulting in high blood pressure and thrombus formation [76]. On the other hand, controversial findings are observed for COX-2 inhibition in humans. Treatment with celecoxib for 1–2 weeks improves endothelial function in hypertensive patients [77], as well as in patients with coronary artery disease [78], while aspirin relieves acute myocardial infarction (AMI) and stroke by inhibiting blood clots [79]; nevertheless, treatment with indomethacin or rofecoxib for 2 weeks failed to improve endothelial function in patients with rheumatoid arthritis [80]. However, other studies show that increased risk of major vascular events is associated with COX-2 inhibitors such as celecoxib and rofecoxib [81,82].

Activation of DP receptors by PGD_2_ induces endothelium- and NO-dependent relaxations in choroidal vessels and increases blood flow, as well as vascular permeability [83]. Importantly, PGH_2_, PGD_2_, PGE_2_, PGF_2α_, PGI_2_, TXA_2_ analog U46619, and 8-isoprostane all induce endothelium-independent contractions in aortas from hypertensive rats through activation of TP receptors [84].

PGE_2_ can act as both vasodilator and vasoconstrictor depending on the receptor subtypes activated: stimulation of EP1 increases intracellular calcium concentration; EP2 and EP4 increase cAMP level, whereas EP3 decrease cAMP level [85]. A previous study has shown that PGE_2_ increases renal vascular tone by stimulating EP1 and EP3, and vice versa, PGE_2_ decreases it by stimulating EP2 and EP4 [86]. PGE_2_ has a relaxing effect in some vascular beds such as human middle cerebral arteries [87] and human pulmonary veins [88] through action on EP4 receptors. On the other hand, PGE_2_ can cause vasoconstriction in some circumstances. PGE_2_ induces contraction in rat mesenteric arteries [89] and human internal mammary arteries [90] via EP3 receptor, and it triggers contraction through concomitant activation of EP1 and EP3 receptors in porcine cerebral arteries [91]. Moreover, the EP1 receptor is involved in the vasoconstriction induced by angiotensin II (Ang II), endothelin-1, and TXA_2_ [92].

PGF_2α_ is a potent vasoconstrictor, increasing blood pressure and atherosclerosis through activation of the FP receptor [93]. Furthermore, PGF_2α_ is shown to trigger endothelium-dependent, TP-receptor-mediated contractions in hamster aortae and human renal arteries; in particular, the impact is increased during aging [94].

TXA_2_ is a potent vasoconstrictor and aggregating factor produced mainly from platelet but also from ECs. The preferential ligand of TP receptors is TXA_2_; nevertheless, TP receptors can be activated to induce contraction by other PGs, isoprostanes, and HETEs at higher concentrations as aforementioned [95].

As aforementioned, free AA is generated upon activation of agonists, such as acetylcholine, and then forms LOX metabolites, such as 12-HETE and 15-HETE, which function as endothelium-derived relaxing factors (EDRFs) in arteries of rodents, rabbits, pigs, dogs, and humans, contributing to relaxation [96]. In addition, 20-HETE mediates contractile response through activation of Rho-kinase [97] and sensitizes vascular SMCs to constrictors such as Ang II, phenylephrine, and endothelin [98]. By contrast, 20-HETE produced by vascular endothelium induces relaxation in pulmonary arteries through activation of eNOS [99].

Extensive evidence supports that EETs formed from AA by CYP enzymes function as EDRF in vascular beds from different species to relax bovine and human coronary arteries [100,101]. Shear stress also activates cytosolic PLA_2_ to liberate AA, which is metabolized to EETs through CYP, and EETs activate TRPV4 channels to increase intracellular Ca^2+^ and subsequently trigger K^+^ efflux from ECs, leading to vasodilation [102].

## 5. Preventing and Managing Vascular Complications in Metabolic Disorders

In long-term streptozocin-induced diabetes, reduced vasodilators PGI_2_ and PGE_2_ and increased vasoconstrictor TXA_2_ contribute to endothelial dysfunction in rat aortas and mesenteric arteries [103]. Accelerated atherogenesis caused by diabetes is associated with increased TXA_2_, and hence, antagonizing the TP receptor by S18886 prevents endothelial dysfunction and atherosclerosis associated with diabetes [104]. Upregulation of COX-2 expression has been linked to impaired cardiovascular function in diabetes and obesity [105]. For instance, palmitate induces endothelial dysfunction through upregulation of COX-2 and the resultant oxidative stress in mouse aortas [106]. Expression of miRNA-200c is found to be elevated in arteries from diabetic mice and patients with diabetes, causing endothelial dysfunction through upregulated COX-2 in ECs and increased generation of PGE_2_ [107]. Downregulation of COX-2 and TP receptor is attributed to the improved renovascular function in estrogen-deficient rats by long-term calcitriol treatment [108].

Notably, 20-HETE is associated with adipogenesis, increasing adiposity and adipocyte differentiation [109], and is elevated in patients with obesity and CVD [110], whereas 12- and 15-HETEs are associated with microvascular dysfunction during diabetic retinopathy [111]. Levels of 12-HETE, 20-HETE, and LTB are increased and closely related to endothelial progenitor cells dysfunction in diabetic patients with cardiac ischemia [112]. Furthermore, inhibition of 20-HETE generation and decreased inactivation of EETs alleviate cardiac dysfunction following ischemic reperfusion injury in diabetes [113]. EET can prevent diabetic cardiomyopathy by maintaining vascular tension, improving myocardial glucose uptake, and reducing related complications caused by diabetes [114]. These findings imply a close linkage between AA metabolites, particularly COX-2 activity, and vascular complications associated with diabetes and obesity.

## 6. Regulation of Blood Pressure

PGE_2_ plays a diverse role in the regulation of blood pressure, determined by the balance between the pressor action of EP1/EP3 receptors and the depressor action of EP2/EP4 receptors. Centrally administered PGE_2_ elevates blood pressure by modulating renal hemodynamics, renin release, and salt and water transport in the nephron, while systemic administration of PGE_2_ generates a hypotensive effect by the diuretic and natriuretic roles in kidney and the depressor action of EP receptors [115,116]. Activation of EP1 receptor by PGE_2_ or the selective agonist increases vascular tone and is responsible for the development of hypertension in diabetic db/db mice [117]. In line with the vasoconstrictive/prohypertensive property of EP1 receptor, EP1 antagonist SC51322 reduces blood pressure in spontaneous hypertensive rats (SHR), whereas genetic disruption of EP1 receptor in mice attenuates AngII-induced hypertension [118]. Both pharmacological inhibition and knockdown of EP3 receptors attenuate pulmonary hypertension [119]. On the other hand, mice lacking EP2 receptors exhibit hypertensive phenotype at baseline and follow a high-salt diet [120]. PGE_2_ relaxes aortic rings and lowers blood pressure in mice by EP4 receptor-mediated stimulation of eNOS activity [121].

In addition to PGE2, PGI2 also plays a significant role in the regulation of blood pressure. Reduced PGI_2_ level contributes to vasoconstriction and platelet aggregation in PAH. Inhalation of PGI_2_ reduces pulmonary artery pressure and pulmonary vascular resistance in patients with residual pulmonary hypertension [122]. Epoprostenol, synthetic PGI_2_ sodium under the name of Flolan^®^ or in the new formulation of Veletri^®^ was the first available drug for treating PAH [123]. The PGI_2_ analogs developed afterward include beraprost, iloprost, treprostinil, and selexipag administered by oral, intravenous, subcutaneous, or inhalation route, improving survival in patients with PAH [124].

COX-2-derived PGF_2α_ impairs endothelial function in renovascular hypertension which can be reversed by treatment with celecoxib (COX-2 inhibitor) [125]. Renal arteries from hypertensive rats and humans show higher expressions of COX-2 and bone morphogenic protein 4 (BMP4). Pharmacological inhibition with COX-2 inhibitor or TP receptor antagonist prevents BMP4-induced endothelial dysfunction in hypertension [126]. Antidiabetic drug sitagliptin has been illustrated to restore endothelial function in SHR and Ang-II-induced hypertensive mice through downregulation of COX-2 and upregulation of uncoupling protein 2 expression in arteries [127]. Studies using several animal models show that 20-HETE mediates eNOS uncoupling and reduces NO production, leading to endothelial dysfunction and hypertension [128,129,130]. These AA metabolites have different functions in regulating blood pressure.

## 7. Antiatherosclerosis Effect

COX-2 inhibitors contribute to atherogenesis owning to favoring the synthesis of TXA_2_ and PGE_2_ while reducing PGI_2_ [131]. Deletion of COX-2 in mice shows accelerated atherogenesis [132]. However, osteocalcin, a skeletal hormone highly expressed in human atherosclerotic lesions, promotes fibroblast transformation through stimulating COX-2 signaling cascade [133]. Ang-II induces COX-2 expression in ECs, which, in turn, increases the generation of proatherosclerotic cytokine monocyte chemoattractant protein-1 [134]. Low-dose aspirin also shows an antithrombotic effect [135]. Inhibition of TXA_2_ by synthetic compound I4 decreases platelet aggregation [136]. Increasing EETs generation inhibits Ang II-induced inflammation and protects against abdominal aortic aneurysms in mice [137].

Similar to the case of hypertension, PGE_2_ plays a crucial role in developing atherosclerosis with diverse actions via different types of EP receptors involved in the formation and stabilization of atherosclerotic lesions. EP4 is the most abundant PGE_2_ receptor expressed in human atherosclerotic lesions, and EP4 overexpression contributes to the deteriorated inflammatory reaction in atherosclerotic plaques [138]. Both genetic and pharmacological inhibitions of EP4 reduce abdominal aortic aneurism formation in mice and humans [139,140]. In contrast to these observations, deficiency of EP4 on bone marrow-derived cells enhances inflammation in atherosclerotic lesions [141] and abdominal aortic aneurism formation [142]. EP2 also implies differential effects in atherosclerosis: its activation promotes the adhesion of monocytes to vascular ECs involved during atherosclerogenesis [143], but genetic disruption of EP2 exacerbates neointimal hyperplasia after arterial injury [144]. Activation of EP3 facilitates atherothrombosis [145], while genetic deletion of EP3 reduces susceptibility to thrombus formation [146] and suppresses neointimal hyperplasia response to injury [147]. Atorvastatin, which suppresses the expression of EP1, EP3, and EP4, effectively protects against thrombotic events [148].

LTB_4_ and LXA_4_, produced by the metabolism of LOXs, are associated with the progression of atherosclerotic lesions. The activation of LTB4 via the 5-LOX pathway can increase the recruitment of neutrophils to atherosclerotic plaques destabilization [149]. LTB_4_ increases vascular permeability by activating the CysLT_2_ receptor in blood vessels, mediating myocardial ischemia and reperfusion injury, and thus, blocking this receptor can alleviate these damages and prevent atherosclerosis [150]. Blocking the binding of LTB_4_ with CysLT receptors helps to stabilize plaque and prevents atherosclerotic lesions [151]. Montelukast (CysLT_1_ receptor antagonist) has been shown to protect heart function and ameliorate atherosclerosis [152]. LXA_4_ is produced by the activation of 15-LOX and has a positive effect on atherosclerosis, in contrast to the destructive effect of LTs [153]. LXA_4_ has effective anti-inflammatory and pro-decomposition abilities, protecting against atherosclerosis [154] and achieving the therapeutic effects of drugs such as aspirin and statins. Aspirin can effectively reduce the plasma level of LXA_4_ in patients with peripheral artery disease and eliminate inflammation [155]. Moreover, lipophilic statins, including simvastatin and atorvastatin, possess strong anti-inflammatory properties, upregulate LXA_4_ expression in the body, and protect the heart function [156].

## 8. Modulating Heart Function and Protecting against Myocardial Infarction

The amount of AA in adipose tissue is found to be associated with the risk of myocardial infarction [157]. Deletion of PGE_2_ synthase-1 accomplished with decreased PGE_2_ level leads to impaired left ventricular contractile function after myocardial infarction [158]. Previous studies have demonstrated the cardioprotective effect of EP3 activation against ischemia/reperfusion (I/R) injury [159,160]. EP3 is necessary for maintaining the normal growth and development of the heart and knockout of EP3 causes eccentric cardiac hypertrophy and fibrosis [161]. Moreover, EP4 is abundantly expressed in the heart with acute myocardial infarction, and activation of EP4 by PGE_2_ or pharmacologically protects the heart from I/R injury [162,163]. Both PGD_2_ [164] and PGI_2_ [165] exert a protective effect on cardiac I/R injury. Low-dose aspirin reduces the risk of myocardial infarction and ischemic stroke [166]. On the other hand, TXA_2_ induces cardiac arrhythmias, and such impairment can be blocked by inhibition of the inositol triphosphate pathway [167]. Receiving combination treatment of aspirin plus atorvastatin, patients with AMI show reduced persistent platelet TXA_2_ production [168].

In addition to PGs, HETEs and EETs affect heart function. Cardioprotective effects of EETs toward acute myocardial I/R injury and cardiac fibrosis have been well reported [169,170,171]. A recent study has shown that AMI patients have a higher baseline level of 20-HETE, which may play a role in the clinical prevention of coronary artery disease [172].

## 9. Clinical Significance of AA Metabolites

AA metabolites have complex modulatory effects upon normal vascular function [9]; therefore, cardiovascular complications may be resulted due to drug treatment-induced alteration of AA metabolism. Aspirin (non-selective COX inhibitor) and celecoxib (selective COX-2 inhibitor) are common nonsteroidal anti-inflammatory drugs (NSAIDs) for treating pain and inflammation [173,174]. Aspirin has been demonstrated to be an effective preventive therapy among patients at risk of developing or suffering from CVD [175,176]. According to American data, aspirin prevents 28% of the risk of coronary heart diseases and 5% of the risk of heart attacks in patients [177]. Although aspirin is cardioprotective, elevated risk of cardiovascular events including myocardial infarction, stroke, hypertension, and congestive heart failure has been reported after using COX-2 inhibitor; in particular, the more COX-2 selective the drug is, the higher is the risk [178]. Therefore, rofecoxib and valdecoxib were withdrawn from the US market [179,180]. Further development of new COX-2 inhibitors is greatly delayed or halted. Celecoxib is still marketed in the US, but individual cardiovascular risk profiles should be evaluated prior to obtaining a prescription [181]. TP antagonists might offset the cardiovascular toxicity of COX-2 inhibitors by blocking TXA_2_ signaling. In turn, inhibitors of TXA_2_ synthase or TP receptors are under development, but no inhibitor has become clinically useful, likely due to the alternative activation of TP by PGH_2_, PGD_2_, PGE_2_, PGF_2α_, or 20-HETE. Evidence from animal studies supports that TP inhibitor terutroban (S18886) slows down the progression of atherosclerosis [182,183]. Despite having no advantage over aspirin, terutroban has similar cardioprotective effects to ischemic stroke, myocardial infarction, or vascular deaths in patients with cerebral ischemic events [184]. Terutroban treatment in patients with a history of ischemic stroke or transient ischemic attack also has a similar protective effect against the progression of carotid atherosclerosis, as compared with aspirin [185]. A dual TXA_2_ synthase inhibitor/TP antagonist EV-077 is better than aspirin to inhibit platelet aggregation in type-2 diabetes [186]. Another possible way to impose a cardiovascular risk of COX-2 inhibitors is to combine with NO or hydrogen sulfide (HS) donors for the provision of additional vasodilatory activity [187]. All these lines of evidence indicate the clinical significance of AA metabolites and that a therapeutic approach targeting AA metabolism can ameliorate the risk factors associated with CVD.

## 10. Potential Health Concerns of Dietary AA

Although our body requires AA, consuming too much of it could be problematic. Israel, one of the countries with the highest level of AA intake, has high prevalence rates of atherosclerosis, diabetes, obesity, and hypertension [188]. This may be, however, confounded by saturated fat and heme iron contained in AA-rich red meat leading to a higher risk of dyslipidemia, thereby an increased risk of CVD [189]. According to a recent survey in Korea, among the highest and lowest consumption of red meat, the risk of dyslipidemia in men increased by 58% and 32%, respectively [190]. A recent Mendelian randomized study has shown that genetically predicted plasma phospholipid AA is positively correlated with atherosclerosis [15]. Moderate consumption of red meat will not increase the risk of CVD, and the nutrients from red meat including AA possess a positive impact on cardiovascular health [191]. Studies have shown that excessive intake of linoleic acid can increase susceptibility to lipid oxidation due to increased plasma low-density lipoprotein (LDL) and diminished high-density lipoprotein (HDL), which may be a major cause of the increased risk of CVD [192]. Moreover, lipid peroxidation alters platelet function and increases the risk of atherosclerosis [193,194]. In fact, replacing saturated fat with omega-6 PUFA has been a cornerstone of dietary recommendations aimed at lowering CVD risk [195]. No upper limit is set for dietary linoleic acid or AA because omega-6 and omega-3 fatty acids counteract the effect of each other and there is a lack of a defined intake found to cause adverse effects [196]. The experimental studies are difficult to translate into clinical research, which involves long-term follow-up investigation of the diet of the individuals and complicated criteria for evaluating dietary intake and timing. In general, AA is beneficial to human health, but excessive intake may cause side effects. Consuming a balanced diet, rather than considering only a single nutrient, is the best choice for human health.

## 11. Conclusions

**Figure 4:**
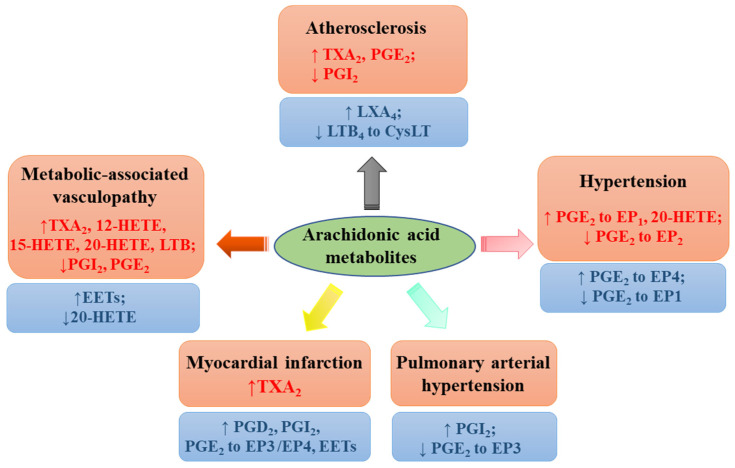
Effects of arachidonic acid metabolites on cardiovascular diseases. Various metabolites including prostaglandin (PG)D_2_ and PGE_2_ (to EP1, EP2, EP3 or EP4 receptor), prostacyclin (PGI_2_), thromboxane (TX)A_2_, leukotrienes B4 (LTB_4_) (to CysLT receptors), lipoxin A_4_ (LXA_4_), hydroxyeicosatetraenoic acids (HETEs), and epoxyeicosatrienoic acids (EETs) exert vasoprotective (labeled with blue color) and deteriorative effects (labeled with red color).

Taken together, extensive lines of evidence imply a diverse and disease-specific contribution of individual AA metabolites to cardiovascular health and complications (Figure 4). More concerted research efforts, especially clinical studies, are required to clarify the controversies and to gain insight into the precise contribution of each AA metabolite targeting specific receptor, aiming at increasing the cardiovascular efficacy of currently available drugs or developing a new class of drugs to overcome CVD.
